# EEG power modulation in the sensorimotor regions is critical to motor tic suppression

**DOI:** 10.3389/fpsyt.2025.1580636

**Published:** 2025-10-28

**Authors:** Makoto Miyakoshi, Joseph Jurgiel, Andrea Dillon, John Piacentini, Scott Makeig, Sandra K. Loo

**Affiliations:** ^1^ Division of Child and Adolescent Psychiatry, Cincinnati Children’s Hospital Medical Center, Cincinnati, OH, United States; ^2^ Department of Psychiatry and Behavioral Neuroscience, University of Cincinnati College of Medicine, Cincinnati, OH, United States; ^3^ Swartz Center for Computational Neuroscience, Institute for Neural Computation, University of California San Diego, La Jolla, CA, United States; ^4^ Semel Institute for Neuroscience and Human Behavior, University of California, Los Angeles, Los Angeles, CA, United States

**Keywords:** chronic tic disorder (CTD), EEG, children, blink, suppression (psychology)

## Abstract

**Background:**

The neural mechanisms underlying tic suppression in chronic tic disorder (CTD) have been investigated using various neuroimaging modalities. A limitation in studying CTD is that abrupt motor action is inherent to the nature of the disorder, but the movement makes any form of neural recording challenging. However, recent advances in hardware and software technologies have enabled EEG studies during motion, which open new avenues for studying CTD with EEG.

**Methods:**

We performed an event-related EEG power spectral analysis in children with chronic tic disorder (CTD) or typically developing children (TDC) as controls in a sample of 76 children (39 CTD) contributing to the final statistics. There were three block-separated conditions: no suppression (NoSupp), suppression with verbal instruction (Supp_Vrb_), and suppression with reward (Supp_Rwd_); the latter two conditions were collapsed into Supp_Ave_. EEG data were processed using independent component analysis, and the event-related potential was decomposed in the time-frequency domain.

**Results:**

During tic or blink suppression, both CTD and TDC showed EEG power increase centered within the theta range in frontal, cingulate, and central regions. Meanwhile, the CTD group showed the opposite pattern in broadband EEG power modulation relative to controls, particularly in the centro-temporal sensorimotor regions. The regression analysis between this broadband power and tic suppression performance resulted in a significant positive correlation.

**Conclusions:**

Better tic suppression was associated with increased EEG power, a similar pattern observed among controls during blink suppression. EEG power in sensorimotor regions is a neural marker of tic suppression performance in children with CTD.

## Highlights

- Children with chronic tic disorder (CTD) performed a tic suppression task. We did not replicate the expected effect of the use of reward for enhancing the suppression.
- We recorded pre-tic brain dynamics and analyzed them using a high-density EEG recording system with advanced signal processing techniques to address the issue of tic-related artifacts.
- We found performance of tic suppression is associated with an increase in EEG power in the right post-central and the left mid-temporal EEG sources.

## Introduction

Tics are defined by sudden, involuntary, and recurrent movements or vocalizations found in children with Chronic Tic Disorders (CTD) including Tourette syndrome. CTD behavior is considered to be related to deficits in motor inhibition and also top-down control. Tics can usually be voluntarily delayed or inhibited on demand, but the neural correlates of this process are still under investigation. A recent meta-analysis found general but inconsistent inhibitory deficits in CTD relative to typically developing controls, in which the effect size varied by task with commonly used inhibitory paradigms include Stop Signal, Flanker, and Go/No-go tasks (1). Clarifying the neural mechanisms of suppression in CTD is important to understand how mental effort controls behavior, and will have critical value in child psychiatry, for example, in designing a clinical behavioral training program for treating children with Tourette’s syndrome.

Neuroimaging studies have shown that the sensorimotor cortex is involved in both the generation and voluntary inhibition of tics. Altered limbic input to the sensorimotor cortex and abnormal GABA-mediated beta-band oscillations measured with magnetoencephalography (MEG) have been detected in the sensorimotor cortex and are suggested to be related to disturbances in sensorimotor processing that may contribute to tic generation (2). Studies using transcranial magnetic stimulation (TMS) have suggested that the primary motor cortex is an important relay in the tic generation circuit, and children with greater tic severity have less excitable motor cortices at rest, possibly as a result of adaptations to suppress unwanted movements (3–5). For the voluntary inhibition of tics, the involvement of the prefrontal cortex has been reported. Regional homogeneity of the blood-oxygen-level dependent (BOLD) signals within the relatively small anatomically defined region within the left inferior frontal gyrus increased in proportion to the CTD children’s ability to inhibit their tics (6). Patients with CTD exhibit increased activation in the direct pathway through the basal ganglia and prefrontal cortex as well as the subthalamic nucleus as compensatory activations (7). These neuroimaging results have high spatial resolution and provide detailed anatomical information related to CTD. However, both positron emission tomography (PET) and magnetic resonance (MR) imaging have general limitations, such as severely affected signal quality due to body movement during scans, and the time course of BOLD changes being on the order of seconds, likely much slower than the timing of neural phenomena. How the intrinsic environment of MR imaging interacts with CTD and its impact on the time resolution of tic events remains unknown. To avoid these limitations in MR imaging, scalp-recorded EEG provides a good alternative.

There are several EEG studies during tic suppression. Prominent increases in theta oscillation in the prefrontal area were observed during tic suppression (8). These authors also reported an increase in partial directed coherence in the frontomotor regions in the theta and beta bands. Elevated alpha coherence was found between sensorimotor areas and the prefrontal and mesial frontal cortex during the suppression of tics (9). However, a decrease in frontotemporal/occipital/parietal connectivity during rest was also reported (10), making the interpretation of the connectivity results somewhat inconsistent. Another study reported employment of the default mode network during tic suppression within the alpha band (11). These EEG studies, as well as most neuroimaging studies, adopted a block design. This is because tic causes body movements that by definition need to be removed from the data analysis, although the target event is the tic itself. Another reason is that EEG research has largely relied on the presence of pre-defined event-related potential (ERP) components, such as P300. In order to fully exploit the richness of high-resolution temporal dynamics of EEG, applying event-related spectral power analysis in the time-frequency domain is desirable. However, event-related EEG analysis targeting the pre-tic interval in children with CTD has not been attempted. An exception is a study reporting peri-tic EEG modulation in adults with CTD (12). However, this pioneering work suffers from poor time-frequency resolution in the result and low validity of in the interpretation of the EEG modulation during tic.

The purpose of the current study is to compare the time-frequency dynamics of event-related EEG during tic and blink suppression between CTD and TDC. This is the first study reporting modulations in broad-band EEG power spectrum during voluntary tic suppression in children with chronic tic disorder. There are two EEG studies on voluntary tic suppression (11, 13), but none of them reported broad-band EEG power modulations in a pediatric cohort. EEG power modulation better translates to localized BOLD signal modulations during tic suppression paradigm (14–16) than phase-based connectivity analysis (11). We expected that the right anterior prefrontal cortex would show power increase based on the previous fMRI studies on tic suppression (16), blink suppression (17), as well as general response inhibition paradigm (18, 19). Our time window of interest is the -5 to 0 seconds prior to the tic or blink event. This approach helps minimize contamination from artifacts caused by body movements. Our group has already established a dedicated signal processing pipeline and has validated its effectiveness with control children (20). Utilizing blink suppression in TDC as an analogue for tic suppression in CTD is an accepted method for studying involuntary movements (16, 21). While eye blinks are a natural phenomenon, they also represent one of the most frequently reported tics (22). Blink suppression paradigms have proven effective for examining the neural correlates of urges in control subjects (17). Finally, we also explored the impact of reward on suppression, as previous research has indicated that reward can enhance successful tic suppression (23, 24). In our earlier study, we found that reward enhanced blink suppression in TDC (20) and we aimed to replicate this finding regarding tic suppression in CTD.

## Methods

### Ethical compliance

The Internal Review Board Committee of the University of California, Los Angeles, approved the current study (IRB number 13 - 00538). The study was conducted in accordance with the provisions of the Declaration of Helsinki. All participants submitted written informed consent. Prior to analysis, we removed participants’ personal information from the data to ensure their de-identification.

### Samples

The children were recruited from the community through social media advertisements, community organizations, and local schools, as well as from the academic medical clinics through primary care physicians and local clinicians. After receiving verbal and written explanations of study requirements, and prior to any study procedures, all parents/participants provided written permission and assent as approved by the Institutional Review Board. A total of 115 children were recruited. In the current study, we focused on those who showed a minimum of 15 tics and blinks during sessions. A subset of 76 children, including 37 typically developing children (TDC, 19 males, mean age 9.6, SD 1.4, range 8 - 12) and 39 children with tic disorder (CTD, 27 males, mean age 9.7, SD 1.6, range 8 - 14), was included in the current study. There was 1 child in CTD comorbid with ADHD on stimulants who was asked to discontinue 24 hours prior to coming into the lab, and 9 children on other psychotropic medicines. The demographic data of this subset is summarized in **Table 1**.

**Table 1:** Demographics and clinical characteristics of participants for the EEG study.

Demographics	TDC	CTD
N	37	39
Age, M (SD)	9.6 (1.5)	9.7 (1.6)
Sex, males, N (%)	19 (51%)	27 (69%)
Full Scale IQ, M (SD)	116 (15)	111 (15)
Clinical Characteristics		
ADHD, N (%)	0 (0%)	23 (59%)
OCD, N (%)	0 (0%)	17 (44%)
Generalized anxiety disorder, N (%)	0 (0%)	12 (31%)
No comorbidities, N (%)	37 (100%)	9 (23%)
YGTSS, M (SD)		
Total score	0 (0)	29.4 (7.7)
Impairment	0 (0)	28.5 (7.9)
PUTS, M (SD)		
Urge presence	N/A	4.7 (2.3)
Urge strength	N/A	4.6 (2.3)
Tic frequency	N/A	5.3 (2.0)
CBCL total, M (SD)	42.0 (9.4)	59.6 (10.3)
SWAN, M (SD)		
Inattention	32.5 (8.5)	23.6 (9.4)
Hyperactivity	32.5 (9.6)	25.6 (9.0)
CYBOCS, M (SD)	N/A	10.0 (10.7)
Medication		
No medication, N (%)	39 (100.0)	29 (74.4)
Stimulant, N (%)	0 (0)	1 (2.6)
Psychotropic, N (%)	0 (0)	9 (23.1)
α -2 agonist	0 (0)	5 (12.8)
Antidepressant	0 (0)	5 (12.8)
Anticonvulsant	0 (0)	1 (2.6)
Antipsychotic	0 (0)	1 (2.6)
Antihypertensive	0 (0)	1 (2.6)

M, mean; SD, standard deviation; IQ, intelligence quotient; ADHD, attention-deficit/hyperactivity disorder; OCD, obsessive-compulsive disorder; YGTSS, Yale Global Tic Severity Scale; PUTS, Premonitory Urge for Tics Scale; CBCL, Child Behavior Checklist; SWAN, Strengths and Weaknesses of ADHD-symptoms and Normal-behavior scale; CYBOCS, Child Yale-Brown Obsessive-Compulsive Scale.

N/A, Not Available.

### Procedures

All participants underwent diagnostic interviews and EEG recording. Psychiatric diagnoses were determined using a semi-structured diagnostic interview, either the Anxiety Disorder Interview Schedule, Child Version (ADIS) (25), modified to cover Tourette and other tic disorders or the KSADS (26) and administered by graduate or doctoral level psychologists. The ADIS and KSADS were supplemented by the clinician-administered Yale Global CTD Severity Schedule (YGTSS) (27), Child Yale-Brown Obsessive-Compulsive Scale (CYBOCS) (28), and Premonitory Urge for CTDs Scale (PUTS) (29). Symptoms of ADHD were measured using the Strengths and Weaknesses of ADHD-symptoms and Normal-behavior (SWAN) scale (30). To assess and quantify broad-band behavioral functioning, parents completed the Child Behavior Checklist (CBCL) (31) and the Behavior Rating of Individual Executive Functions (BRIEF) (32). Senior clinicians (JP, SC, AD) confirmed the presence of DSM - 5 psychiatric diagnoses after an individual review of each participant’s symptoms, duration, and impairment level. Estimated intelligence (IQ) was assessed using the Wechsler Abbreviated Scale of Intelligence (WASI) (33). Subjects were excluded from participation if they were positive for any of the following: head injury resulting in a concussion, diagnoses of autism, major depression, bipolar disorder, panic disorder or psychosis, estimated Full Scale IQ < 80 or YGTSS <15 (CTD only). In addition, TDC subjects were excluded if they had any major Axis I diagnosis or were on any type of psychoactive medication.

### Task

There were three block-separated conditions: tic or blink freely i.e., no suppression (NoSupp), verbal instruction for blink suppression (Supp_Vrb_), and blink suppression for reward (Supp_Rwd_). All children were instructed to tic or blink freely during the NoSupp block, while trying to suppress tics/blinks during the two suppression blocks. During Supp_Rwd_, children were told that the computer would be counting how many tics/blinks they were able to suppress, and that they would subsequently receive a reward for successful suppression. All children received $10 regardless of how many tics or blinks they exhibited. The order of the three conditions was counterbalanced across subjects. Each block length was between 5 – 7 min. This variability is due to the update of the experimental protocol in the middle of the project.

### Identifying tics and blinks

For annotating tics, we used the video recordings that were timestamp-synchronized to EEG and other data streamed by LSL to identify the onset of the tics. There was a single rater to annotate tic onset markers. For annotating blinks, we developed an EEGLAB plugin called *countBlinks()* for this project (available from https://sccn.ucsd.edu/eeglab/plugin_uploader/plugin_list_all.php) to manually annotate all the blinks during the tasks by visually examining the time-series data of the independent component (IC) representing blink/vertical eye movement. For a detailed description and validation of this program, see Miyakoshi et al. (20).

### EEG recording

EEG signals were recorded using the Electrical Geodesics Incorporated (EGI) hardware and software with 128 Hydrogel electrodes embedded in a hydrocel net in an International 10/10 location system. The data were sampled at 1000 Hz and initially referenced to Cz. The electrode-skin impedance threshold was set at 50 kOhms per the manufacturer’s standard for the high input impedance amplifier. Eye movements were monitored by electrodes placed on the outer canthus of each eye for horizontal movements (REOG, LEOG) and by electrodes above the eyes for vertical eye movements. Facial electromyography (EMG) leads were placed on the cheeks bilaterally over the zygomaticus major muscles to assist with the detection of facial movements. Key head landmarks (nasion, inion, preauricular notches) and 3-D electrode locations were recorded (Polhemus, Inc.) to allow for the reconstruction of electrode positions on the scalp. All EEG data were recorded using the Lab Streaming Layer (LSL, https://github.com/sccn/labstreaminglayer), which allowed for the integration of multiple data streams including EEG, high-definition video, and experimental events.

### EEG preprocessing

Throughout the preprocessing, we used EEGLAB 14.1.2 (34) running under Matlab 2017b (The MathWorks, Inc., Natick, MA, USA). Custom code was written as necessary. There were two central signal processing techniques: artifact subspace reconstruction (ASR) (35–41), which is an offline version of data cleaning suits from BCILAB (42), and independent component analysis (ICA) (43–46). The details of the method can be found in our previous publication (20). These two approaches are complementary in that ASR uses sliding-window principal component analysis (PCA)-based subspace rejection and reconstruction so that it can address data non-stationarity such as infrequent short-lasting bursts by touching electrodes, for example, while ICA can find stationary processes and temporally maximally independent sources such as brain EEG sources as well as non-brain artifact sources like blink, eye movement, and facial and neck muscle activation by using more sophisticated, physiologically valid assumptions than PCA (47, 48). For each of the obtained independent components (IC), an equivalent current dipole model was fitted using Fieldtrip function dipfit3.3 (49) and fitTwoDipoles (50). The obtained ICs were also automatically annotated by ICLabel (51) to preselect only ICs labeled as ‘Brain’. The mean rate of electrode rejection rate was 2.8% (SD 1.9, range 0 - 9.4) and 5.3% (SD 2.1, range 4.1 - 16.4) for control and children with tic, respectively. The mean datapoint rejection rate was 0.67% (SD 2.1, range 0 - 11.4) and 0.4% (SD 0.9, range 0 - 4.1) for control and children with tic, respectively. After performing the IC rejection, the average number of ICs submitted to the final analysis was 48.4 (SD 11.2, range 16 - 68) and 40.1 (SD 13.0, range 18 - 65) for control and children with tic, respectively.

### Group-level analysis

The prototype of the EEGLAB plugin *Eyen* was used to perform the following group-level analysis. A total of 3470/9312 ICs (37.3%) with a ‘Brain’ label were collected from the preselected 76 participants and submitted to k-means clustering using the dipole locations. The Silhouette Index (52) determined that the optimum number of clusters was 13. We analyzed event-related spectral perturbation (ERSP) on each anatomically associated IC cluster to investigate time-frequency-space decomposed EEG power dynamics related to tic or blink suppression.

### Statistical testing

For the behavioral data analysis, the factorial design for the tic or blink count analyses was 2 (Groups: CTD and TDC) x 3 (Suppression Conditions: NoSupp, Supp_Vrb_, Supp_Rwd_). A paired t-test was performed across all the pairs of Suppression Conditions within each Group. Because the behavioral difference between Supp_Vrb and Supp_Rwd_ failed to show significance, we collapsed these conditions into a single Supp_Ave_ condition in the following analyses. To study the relationship between premonitory urges and tic frequency, we conducted correlation analyses between PUTS scores and normalized tic frequencies in NoSupp and Supp_Ave_ conditions.

For the EEG data analysis, the factorial design was reduced to 2 (Groups: CTD and TDC) x 2 (Suppression Conditions: NoSupp and Supp_Ave_). A mixed design ANOVA was performed on each time-frequency cell of the calculated ERSP tensor with the dimensions of 50 (logarithmically spaced frequency bins, 1 to 50 Hz) x 1251 (latency to tic or blink, -5000 to 0 ms) x number_of_ICs (this varies from IC cluster to cluster) for 13 IC clusters. For the multiple comparison correction for the 50 x 1251 time-frequency points, weak family-wise error rate (wFWER) control was used (53). *F*-statistics values were computed for all time-frequency points and thresholded at *p* < 0.05. The true *mass of the cluster*, which is the sum of absolute *F*-statistics within a time-frequency pixel cluster, was computed for each time-frequency pixel cluster separately. Next, the data labels were shuffled and the same procedure was applied, and the largest *mass of the cluster* was taken to build a distribution of surrogate *mass of cluster*. Finally, the 95th percentile of the surrogate *mass of the cluster* distribution was obtained to determine the threshold value for omnibus correction. Those true *mass-of-cluster* entries that were larger than the threshold value were declared to be statistically significant after wFWER control.

In order to study the relationship between behavioral performance in suppression and EEG power modulation, the suppression ratio was defined and calculated for all participants.


$$\text{Supression Ratio}=\text{NoSupp}/(({\text{Supp}}_{\text{Vrb}}+{\text{Supp}}_{\text{Rwd}})/2)$$

We calculated the linear correlation between ΔERSP, which is defined as the ERSP difference (Supp_Vrb+Supp_Rwd)/2−NoSupp , and the Suppression Ratio for each IC cluster. When multiple ICs were associated with a single subject, the mean value was calculated across the ICs. To obtain the variance of this correlation, a bootstrap test was performed using the Matlab bootstrp() function with 2000 iterations on ΔERSP, and the linear correlation against the Suppression Ratio was calculated. In addition, we also conducted correlation analyses between ΔERSP and PUTS scores to examine the relationship between pre-tic EEG modulations and premonitory urges.

Finally, dipole density was generated by convolving a 3-D Gaussian smoothing kernel with FWHM = 20 mm. The Talairach atlas was used to determine the three highest associated anatomical labels (54, 55).

To control for the effects of age and drug (any psychotropic drug other than stimulants, which were discontinued 24 hours prior to testing), we utilized a linear mixed effect model (LME) implemented in Matlab using the fitlme() function. The formulae used for testing for group differences in the blink/tic counts and EEG power, as well as the within-group regression analysis between tic suppression score and EEG power, respectively, were as follows


Counts of blinks/tics or EEG power = Age + Drug + Condition*Group

EEG power = Age + Drug + Suppression Score

where EEG power represents a numerical vector containing EEG’s ERSP power within the statistically significant masks in the time-frequency domain, Age is a numerical vector indicating the participants’ ages at the time of recording, Drug is a categorical variable denoting the use of psychotropic medication, Conditions is a categorical variable consisting of {NoSupp, Supp_Vrb_, Supp_Rwd_}, and Group is a categorical vector comprising {CTD, TDC}. All of these were fixed effect variables. We did not include random effect variables for adopting the same fixed-effect statistics between behavioral and EEG data. Applying an LME model including a random effect for all time-frequency calculations would take 40.6 days to finish based on our estimation, which was not practical. We did, however, test the random intercept in the behavioral data: a likelihood-ratio test comparing the fixed-only model with a mixed model (σ = 3.63, Δχ² = 65.9, df = 1, p = 4.4 × 10^−16^) showed a better fit but did not change any significance decisions. Hence the simplified model is inferentially equivalent while remaining computationally feasible. We reported uncorrected p-values for these tests.

## Results

### Demographics

Demographic data for the primary study sample as well as the classifier test sample are described in **Table 1**. The primary sample displayed moderate tic severity with a mean total YGTSS score of 29.4 ± 7.7. Comorbidities of ADHD, OCD, or an anxiety disorder were present in 77% of participants, as is typical for such populations.

### Behavioral data

**Figure 1:**
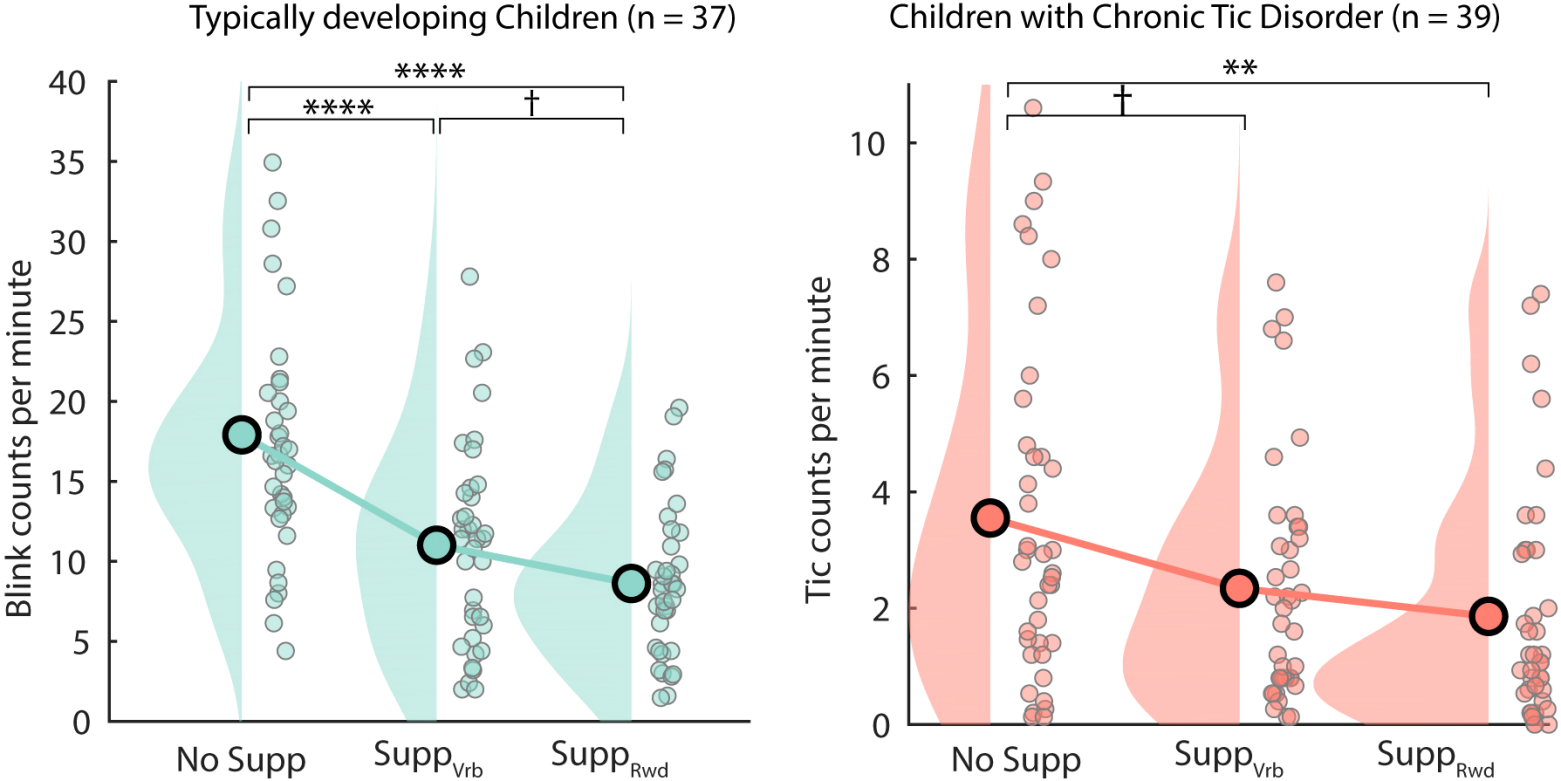
The number of blinks and tics per minute for TDC (left) and CTD (right), respectively. For both groups, the effort to suppress the blinks and tics was effective. Note that the effect of using reward compared with no reward was marginal for TDC and non-significant in CTD. ^†^p < 0.10; **p < 0.01; ****p < 0.001. p-values reported here are corrected with False Discovery Rate.

The numbers of tics and blinks were counted for each block and normalized into average counts per minute for each subject. The results are shown in **Figure 1**. For TDC, NoSupp, M = 17.9 (SD 8.8, range 4.4 - 48.6); Supp_Vrb_, M = 11.0 (SD 6.3, range 2 - 27.8); Supp_Rwd_, M = 8.6 (SD 4.7, range 1.5 - 19.6). For CTD, NoSupp, M = 3.6 (SD 2.9, range 0.1 - 10.6); Supp_Vrb_, M = 2.3 (SD 2.0, range 0.1 - 7.6); Supp_Rwd_, M = 1.9 (SD 2.0, range 0 - 7.4). The planned comparisons revealed significant differences between NoSupp *vs*. Supp_Rwd_ for both groups, both *t* (72) and *t* (76) > 2.99 for TDC and CTD, respectively, all *P* < 0.01 (FDR corrected). The differences between NoSupp *vs*. Supp_Vrb_ were significant for TDC, *t* (72) = 3.88, *P* < 0.001, but it was marginal for CTD, *t* (76) = 2.12, *P* = 0.056. The difference between Supp_Vrb *vs*. Supp_Rwd_ was marginal for TDC, *t* (72) = 1.85, *P* = 0.082, and non-significant in CTD, *t* (76) = 1.05, *p* = 1. Because the behavioral results did not confirm the clear difference between Supp_Vrb *vs*. Supp_Rwd_, these two conditions were collapsed hereafter.

In order to assess the impact of Age and Drug on the result, we conducted LME modeling and included Age and Drug as fixed effect variables. The goodness of fit was evaluated using R^2^. The adjusted R^2^ was 0.526. Neither Age nor Drug showed any significant impact (*p* = 0.649 and 0.912, respectively), while all other factors and the interaction term were found to be significant (p < 2.81 x 10^-6^). We conclude that Age and Drug cannot be dominant factors in the behavioral data.

The correlation analyses between PUTS scores and the normalized tic counts in NoSupp and Supp_Ave_ conditions, as well as the difference NoSupp - Supp_Ave._ None of the tests reached statistical significance: *R* = 0.158 (*p* = 0.181), *R* = 0.147 (*p* = 0.214), and *R* = 0.055 (p = 0.643), respectively. Thus, our data did not support the prediction that premonitory urge correlates with tic severity (56).

### EEG data: general description

37 TDC datasets showed an average of 48.4 ICs (SD 11.2, range 16 - 68), while 39 CTD datasets showed an average of 40.1 ICs (SD 13.0, range 18 - 65). The Silhouette algorithm determined the optimum number of k-means clusters as 13 (52). The mean number of TDC datasets and ICs per cluster is 34.8 (SD 2.1, range 30 - 37) and 142.3 (SD 36, range 84 - 198), respectively, and for CTD datasets, it is 35.9 (SD 4.1, range 24 - 39) and 124.6 (SD 35.5, range 51 - 181).

### EEG data: main effect suppression

**Figure 2:**
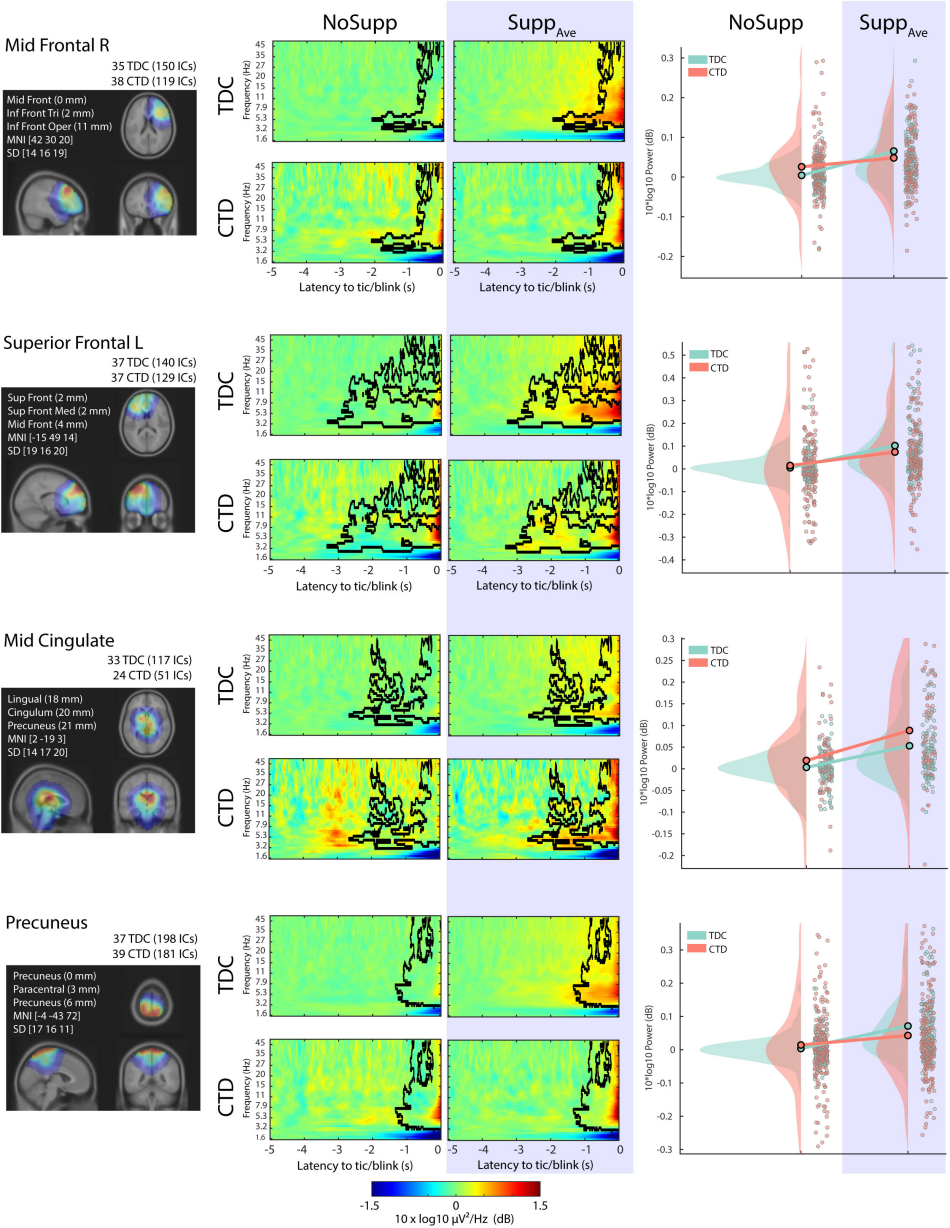
The four IC clusters that showed a main effect of Suppression, all of which showed a power increase during suppression. Left: The relative dipole density after applying 3-D Gaussian smoothing with FWHM 20 mm. Middle: The 2x2 event-related spectral perturbation (ERSP) plots. The baseline period was defined between -5 to -4 s relative to blink/tic onset. The black contour indicates the significance mask at p < 0.05 corrected for cluster-level family-wise error-rate (FWER) control. Right: The scatter plot and distribution of the mean value within the significance mask across ICs. TDC, typically developing children; CTD, chronic tic disorder.

Out of the 13 IC clusters, 4 of them showed the main effect of Suppression, including 2 frontal clusters (**Figure 2**). These frontal IC clusters, Mid Frontal R and Superior Frontal L, are characterized by the long-lasting theta-band power increase for Supp that started -3 to -2 s before the blink/tic onset. Our previous study reported frontal EEG power increase during blink suppression in typically developing children (20). The current result confirms that the same frontal EEG power increase also occurs in the CTD population during tic suppression. The result also confirms the view that blink suppression can be an analogue of tic suppression in terms of the frontal EEG power modulation. We also found that the Mid Cingulate IC cluster also showed the similarly extended pre-blink/tic power EEG power increase occurred in both groups, indicating functional correlation.

Note that the current dipole density of the Mid Cingulate IC cluster covering midbrain regions is physiologically implausible as a current source for the EEG signals. These deep current dipole models are results of the depth bias in fitting a *single* dipole model to a scalp topography contributed by a broad dipole *layer.* A typical dipole layer consists of a massive parallel array of micro dipoles that covers substantial (> 6.45 cm^2^) area of the cortical surface (57). The impact of Age and Drug was evaluated using the LME model including Age and Drug as fixed effect variables. For all the IC clusters shown in **Figure 2**, the main effect Suppression remained significant (all *p* < 6.17x10^-6^) after separating the effect of Age and Drug. The effect of Age was significant for the superior frontal (*p* = 0.00254) and the precuneus (*p* = 0.00035) IC clusters. The effect of Drug was significant for the precuneus (*p* = 6.29x10^-6^). The adjusted R^2^ ranged from 0.0554 to 0.111.

### EEG data: interaction Suppression x Group

**Figure 3:**
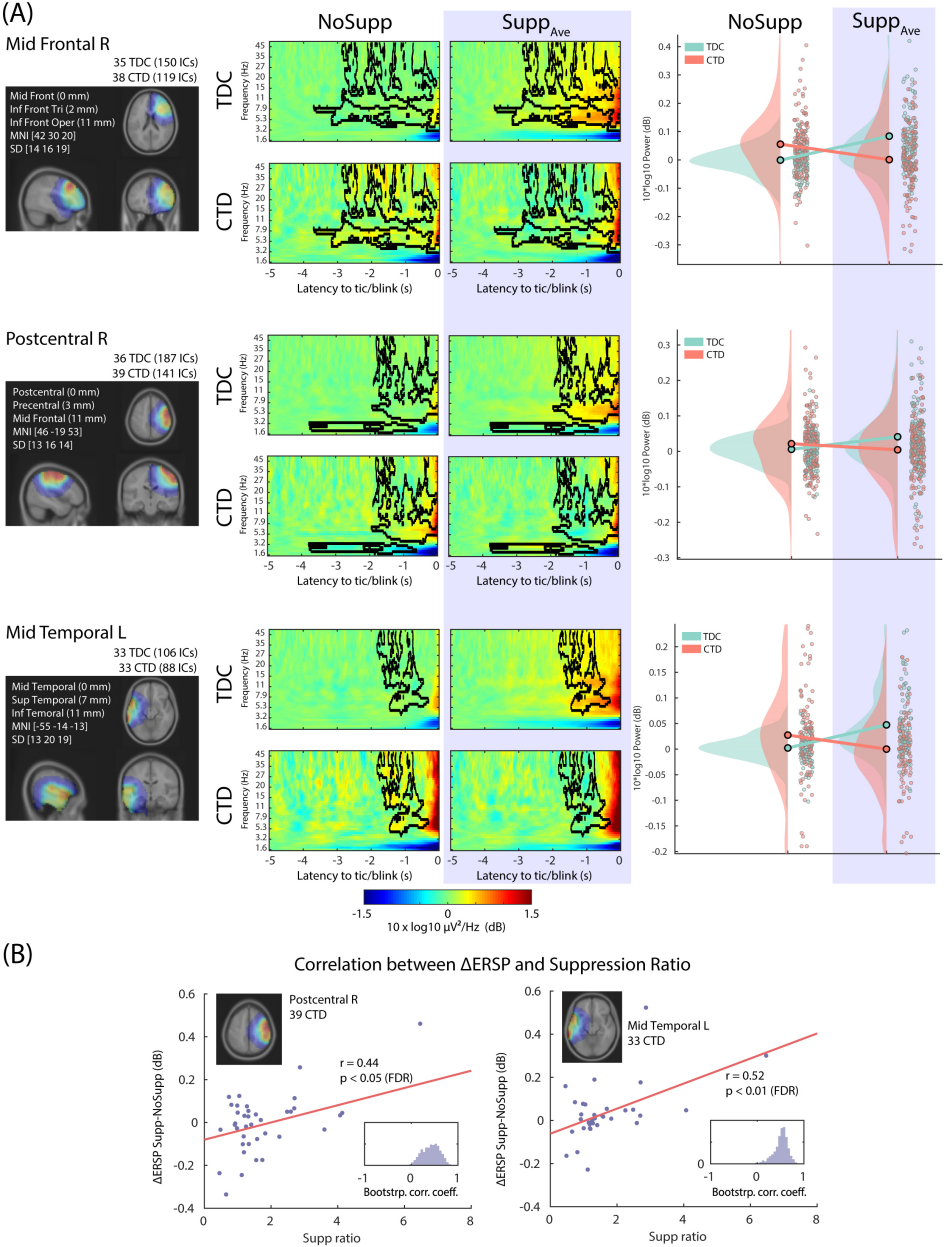
The three IC clusters that showed interaction Suppression x Group, all of which showed suppression-related power decrease in CTD. **(A)** Left, the relative dipole density after applying the 3-D Gaussian smoothing with FWHM 20 mm. Middle, the 2x2 event-related spectral perturbation (ERSP) plots. The baseline period was defined between -5 to -4 s relative to blink/tic onset. The black contour indicates significance mask at p < 0.05 corrected for cluster-level family-wise error-rate (FWER) control. Right, the scatter plot and distribution of the mean value within the significance mask across ICs. **(B)** For the right postcentral and the left precentral/temporal IC clusters, the performance score of tic suppression (the horizontal axis, the higher the better) showed a positive correlation with relative EEG power increase during tic suppression compared with no suppression condition. The EEG power increase in these regions is the characteristic of control groups during blink suppression. TDC, typically developing children; CTD, chronic tic disorder.

Out of the 13 IC clusters, 3 of them showed a significant interaction effect Suppression x Group, including 1 frontal and 2 central/temporal clusters (**Figure 3**). The right prefrontal IC cluster, which showed a similar power increase in both groups mainly in the theta-alpha range, now showed the opposite pattern in the beta range. These differential EEG power modulations across frequencies between the groups suggest the presence of abnormal beta-gamma EEG power modulation in the CTD. A similar pattern was found in the right central and left centro-temporal regions in broadband power (4 to 45 Hz), which indicates abnormal EEG power modulation in the bilateral sensorimotor regions.

The impact of Age and Drug was evaluated using the LME model including Age and Drug as fixed effect variables. For all the IC clusters shown in **Figure 3**, the interaction Suppression x Group remained significant (all *p* < 0.00166) after separating the effect of Age and Drug. The effect of Age was significant for the postcentral (*p* = 0.00211) IC cluster. The effect of Drug was significant for the postcentral (*p* = 0.000294) and the temporal (*p* = 0.000682). The adjusted R^2^ ranged from 0.0426 to 0.0986.

Subsequently, we performed correlation analyses between the EEG power and the suppression performance. After applying the FDR correction across all the IC Clusters x Groups, only CTD in 2 IC clusters showed a significant result: better suppression in CTD was associated with increased power (i.e. °ΔERSP): *r* = 0.44 and 0.52 for the right central and the left centrotemporal ICs, respectively (*p* < 0.05 and *p* < 0.01, respectively, after FDR correction). Because TDC showed increased power during suppression, we interpreted the result that children with CTD with better suppression also showed more similar EEG power modulation to the TDC. We used the LME model to evaluate the impact of Age and Drug. For both IC clusters, the results from the correlation analysis remained significant (both *p* < 0.00618). The effect of Age did not reach significance (all *p* > 0.359). The effect of Drug was significant (*p* = 0.0406 and 0.0408, respectively) but would not survive multiple comparison corrections.

Finally, we conducted correlation analyses between EEG power (NoSupp, Supp_Ave_, and ΔERSP i.e., NoSupp-Supp_Ave_) and PUTS scores for each IC cluster. None of the results survived multiple comparison corrections using FDR. However, two IC clusters showed significant results only in ΔERSP in uncorrected p-values: the left superior frontal cluster (**Figure 2**, the second row) showed *r* = 0.357, *p* = 0.028, and the left parieto-occipital cluster (not shown) showed *r* = 0.427, *p* = 0.009. These results may indicate preliminary EEG evidence of a link between premonitory urge and suppression-triggered EEG modulations.

## Discussion

We performed independent component analysis (ICA)-decomposed event-related potential analysis in the time-frequency domain to study brain dynamics during suppression of tic or blink using CTD and TDC subjects. We found three main results: (1) The main effect of Suppression was found in mostly frontal but also central regions reflected by power increase in the broad frequency band including the theta range, which indicates a common EEG signature between both groups; (2) an interaction effect of Suppression x Group was found in centro-temporal sensorimotor regions (in the broadband), as well as a frontal region (in the beta and gamma range) in which CTD showed power decrease while TDC showed power increase. Notably, there was a positive correlation between EEG power and performance in behavioral suppression in CTD, indicating that good suppressors in CTD show EEG power increase similar to TDC; (3) The effect of reward on tic suppression behavior was not significant.

### Main effect of suppression

The main effect Suppression in both CTD and TDC was represented by an EEG power increase mainly in the theta band. We recently reported prefrontal EEG power increase during blink suppression in typically developing children (20). The current result extends the finding that this prefrontal theta power increase is probably a common EEG signature for tic suppression in CTD. The involvement of prefrontal regions, including the dorsolateral prefrontal cortex (DLPFC), in voluntary non-tic-related inhibition tasks (18, 19) as well as a tic inhibition task (16) confirms that our ICA-derived EEG sources activation in the time-frequency domain is in line with these neuroimaging studies. Thus, our results confirmed the expected engagement of the right prefrontal region in the task of tic suppression.

Another important region that showed up was the cingulate cortex. Care must be taken when interpreting the location of the IC cluster: the apparent location of the cluster centroid is in the subcortical region, but this is obviously due to the depth bias as mentioned before. When fitting a single equivalent current dipole model to a broad cortical source, the estimated location becomes deeper than the actual depth of such a layer of a dipole array. Distance-dependent attenuation of electric potential produced by a single dipole follows the inverse square law. However, the rate of the distance-dependent attenuation can be substantially mitigated by forming a widespread parallel dipole array (for more details, see Supplement 2 of Miyakoshi et al., (106)). Thus, we include a broader area of the cingulate cortex, including a posterior division of the anterior cingulate cortex (ACC) for the result interpretation. ACC has been associated with various types of urges such as itch (58), voiding of urine (59, 60), coughing (61, 62), and smoking (63). ACC is involved in subjective feeling, response coordination, self-monitoring, assessment of motivational valence, and initiation of motor actions (64). ACC has been known to be a region where regulatory and executive processes interact (65). Involvement of ACC was also reported in a previous blink suppression study (66) and anti-saccade study (67). One of the above cited studies concluded that bilateral dorsolateral prefrontal cortices and the anterior cingulate cortex are the key regions for suppression tasks for both children and adults (68), which is in line with the current result.

The anatomical and functional connection between the ACC and insula should be mentioned. Note that we do not intend to make a claim that any of our EEG results directly reflect insula activity, since epilepsy research indicated that scalp-recorded EEG cannot directly measure insula (69–72). However, it is obvious from neuroimaging studies that the insula is another central region involved in various kinds of suppression tasks, including blink (16, 17, 66, 73), air hunger (74), cough (75), and even the stop signal task in CTD (76). Both the ACC and insula are known to contain von Economo neurons (77) which are particularly large neurons unique to these regions and only found in great apes and humans. There is anatomical evidence that Brodmann area 24 has a reciprocal connection with the insular cortex (78, 79), which may be mediated by the von Economo neurons (80). There is additional evidence that the insula and midcingulate cortex, which represent limbic sensory and motor regions respectively, are the key network for motivation for action in urge (81). These M/ACC-insula connections may indicate an underlying anatomical structure that is indirectly reflected by the theta-band power increase we found in both groups in the cingulate cortex.

### Interaction between suppression and group

In comparing CTD and TDC in tic or blink suppression, we found a group difference in the broadband power in the frontal and centro-temporal regions. We interpreted this centro-temporal area as representing sensorimotor regions. The involvement of the frontal region is discussed above, but notably this group-difference was found in the beta band. This may suggest there are differential EEG power modulations in non-overlapping frequency ranges between the groups: theta power changes are common, but beta power changes are the opposite between CTD and TDC. The involvement of sensorimotor regions in CTD has been reported in previous EEG studies (9, 82, 83). It is hypothesized that the inhibitory control exerted by frontal structures, such as the dorsolateral prefrontal cortex, inferior frontal gyrus, and supplementary motor area during voluntary movement and self-regulation, may be consistent with neural activity reported during tic inhibition and suppression (6, 84, 85). Furthermore, altered states and activities of the sensorimotor cortex in CTD were also reported by an MEG study (2) and TMS studies (3–5). These frontal and sensorimotor locations reported in the study of CTD are consistent with our findings. Furthermore, for the EEG power resolved at the bilateral sensorimotor areas, our results revealed that those children with CTD who could suppress the tics better showed greater increase in broadband power although the group tendency of CTD is the broadband power decrease. We interpret that the broadband power increases in the bilateral sensorimotor regions can be a neural marker that reflects the CTD children’s ability to suppress tic. The time window of this broadband EEG modulation is about -2 to -1 sec until the suppression breaks and the movement begins. The time-frequency parameters of this important EEG marker were for the first time resolved spatiotemporally thanks to the EEG event-related potential paradigm.

### The effect of reward in CTD

The facilitation of blink suppression by reward was only marginal in typically developing controls (TDC; *p* = 0.074) and absent in CTD (*p* ≈ 1). This difference suggests reduced reward sensitivity in CTD. One likely reason is that, in our experimental design, rewards were delivered late and in bulk, rather than immediately and repeatedly at short intervals. The dopaminergic hyper-innervation hypothesis of CTD (86, 87) predicts that an elevated tonic dopamine level in CTD diminishes the impact of delayed, but less so for immediate, rewards. Consistent with this view, Greene and colleagues found that when children with CTD received a contingent reward after each 10-s tic-suppression block, performance improved markedly, whereas yoked, non-contingent rewards had little effect (23). Although we informed participants that the reward was contingent, its bulk, delayed delivery likely made it less effective for CTD. Thus, our interpretation is that elevated tonic dopamine level blunted the reward signal for bulk and delayed delivery in CTD.

Another possibility is that the effect of the EEG recording environment, such as wearing an electrode cap, may have disrupted tic and tic suppression behaviors and changed the CTD group’s sensitivity to reward. Finally, the low base rate of tics in the NoSupp condition may have created a floor effect that negatively impacted the amount of decrease that was possible during the Supp_Rwd_ condition.

### Relation to neural network dysfunction associated with CTD

A translational study in transgenic mice demonstrated that a hyper-glutamatergic cortico-striato-pallido-thalamo-cortical (CSTC) loop can produce Tourette-like tics (88). In primates, the dorsal striatum, i.e., the putamen, receives dense glutamatergic input from frontal sensorimotor cortices (89–93). According to the hyper-glutamatergic CSTC model, excessive pyramidal drive from the motor cortex reaches the putamen (94–97), where more than 90% of neurons are GABAergic medium spiny neurons that project to the globus pallidus and substantia nigra (98). These output nuclei inhibit the anterior ventrolateral thalamus, whose efferents return to anterior M1 (99), completing a dysregulated feedback loop that favors tic generation. Human MRI work corroborates this circuitry, revealing putaminal and thalamic structural and tractographic abnormalities in CTD (100, 101). Consistent with these findings, our results identified a right post-central sensorimotor component (**Figure 2**) whose activity scaled with voluntary tic suppression, and a mid-cingulate component that overlaps regions where gray-matter volume correlates with premonitory urges (100). Although scalp EEG cannot directly capture sub-cortical signals, anchoring analyses in the hyper-glutamatergic CSTC framework enables theory-driven interrogation of cingulate and sensorimotor oscillations as cortical read-outs of basal-ganglia dysfunction.

### Implications for behavioral treatment

Comprehensive behavioral intervention for tics (CBIT) (102, 103) builds on habit reversal training (104, 105). Its success depends on trainee’s ability to detect pre-monitory urges and to execute a voluntary counter-action, a process that recruits cingulo-sensorimotor circuits. Our data show recruitment of the mid-cingulate and sensorimotor cortices during instructed tic suppression, which are the key cortical nodes both for the CSTC model and the CBIT. These cortically contributed EEG signatures may serve as objective markers of therapeutic engagement. Future studies should examine whether CBIT modulates these EEG markers for normalization. Until such evidence is available, the present findings provide physiological support for the neural rationale underlying CBIT/HRT.

### Limitations

We studied children with CTD, who may not be representative of adults with CTD due to potential developmental adaptations. Care must be taken when generalizing our results to adults with CTD. The same limitation applies to the difference between motor tics, which we focused on in this study, and vocal tics. Because of potential differences in neural mechanisms between the two types of tics, appropriate limitation must be added when generalizing the current results to the case of vocal tics.

We could use only about half of the CTD samples because the other half of the data sets did not meet inclusion criteria of minimum 15 trials per condition for 3 conditions. This could have excluded good tic suppressors from the final EEG analysis. When interpreting the obtained EEG results, we must take into consideration that the CTD data sets are biased towards poor suppressors. Finally, the gender balance in the CTD sample is biased towards male (27 males *vs*. 12 females). The effect of the interaction between CTD and gender imbalance is unknown. When interpreting the current results, this limitation should be considered.

## Conclusion

We performed the first event-related potential analysis targeting suppression breaks to study the brain dynamics of the pre-tic or blink period. We found that both the CTD and TDC showed increased EEG power centered in the theta range in frontal, cingulate, and central regions. Meanwhile, we found that CTD showed a reversed pattern in broadband EEG power modulation in the centro-temporal sensorimotor regions. The regression analysis between this broadband power and suppression performance indicated a positive correlation in CTD, indicating that better tic suppression was associated with increased EEG power, similar to the TDC group during blink suppression. Thus, EEG power is a putative neural marker of tic suppression performance in CTD.

## Data Availability

The data that support the findings of this study are available from the senior author, SKL (Sloo@mednet.ucla.edu), and Matlab code is available from the corresponding author, MM, upon reasonable request following the completion of all necessary IRB-related registrations and approvals for data sharing.
